# Dormant phages of *Helicobacter pylori* reveal distinct populations in Europe

**DOI:** 10.1038/srep14333

**Published:** 2015-09-21

**Authors:** F. F. Vale, J. Vadivelu, M. Oleastro, S. Breurec, L. Engstrand, T. T. Perets, F. Mégraud, P. Lehours

**Affiliations:** 1 Université de Bordeaux, Laboratoire de Bactériologie, Bordeaux, France; 2 INSERM U853, Bordeaux, France; 3 Host-Pathogen Interactions Unit, Research Institute for Medicines (iMed-ULisboa), Instituto de Medicina Molecular, Faculdade de Farmácia da Universidade de Lisboa; 4 UM Marshall Centre and Dept of Medical Microbiology, University of Malaya, Lembah Pantai, 50490 Kuala Lumpur, Malaysia; 5 Laboratório Nacional de Referência das Infeções Gastrintestinais, Departamento de Doenças Infeciosas, Instituto Nacional de Saúde Dr Ricardo Jorge, 1649-016 Lisboa, Portugal; 6 Institut Pasteur, Laboratoire de Bactériologie, Bangui, République Centrafricaine; 7 Institut Pasteur, Laboratoire de Bactériologie, Dakar, Senegal; 8 Department of Microbiology, Tumor and Cell Biology, Karolinska Institute, Stockholm, Sweden; 9 Gastroenterology Laboratory, Rabin Medical Center – Beilinson Hospital, Petah Tikva, Israel; 10 Sackler Faculty of Medicine, Tel Aviv University, Tel Aviv, Israel

## Abstract

Prophages of *Helicobacter pylori*, a bacterium known to co-evolve in the stomach of its human host, were recently identified. However, their role in the diversity of *H. pylori* strains is unknown. We demonstrate here and for the first time that the diversity of the prophage genes offers the ability to distinguish between European populations, and that *H. pylori* prophages and their host bacteria share a complex evolutionary history. By comparing the phylogenetic trees of two prophage genes (integrase and holin) and the multilocus sequence typing (MLST)-based data obtained for seven housekeeping genes, we observed that the majority of the strains belong to the same phylogeographic group in both trees. Furthermore, we found that the Bayesian analysis of the population structure of the prophage genes identified two *H. pylori* European populations, hpNEurope and hpSWEurope, while the MLST sequences identified one European population, hpEurope. The population structure analysis of *H. pylori* prophages was even more discriminative than the traditional MLST-based method for the European population. Prophages are new players to be considered not only to show the diversity of *H. pylori* strains but also to more sharply define human populations.

To discover and follow human migrations, the use of information from genomes of human pathogens and commensals which present a phylogeographic division, may offer additional insight into the corresponding human genome sequences themselves. *Helicobacter pylori* and man co-evolved together, since they went ‘out of Africa’^1^,^2^. This bacterium colonizes the human stomach of more than half of the human population. The infection is not without a clinical impact, and although the majority of the human hosts do not present any symptom, gastritis is present in all cases. Complications of gastritis include peptic ulcer disease and, in rare cases, gastric adenocarcinoma and mucosa associated lymphoid tissue (MALT) lymphoma^3^.

The co-evolution of the bacteria with the human host is verified by phylogenetic analysis which produces bacterial clusters according to the geographic origin of the bacterium and its host (reviewed in^4^,^5^). Currently, 7 *H. pylori* bacterial populations have been described, following MLST analysis of 7 housekeeping genes^2^,^6^ but only one European population, hpEurope, is considered here.

Bacteriophages (phages) are viruses which infect bacteria. Lytic phages have the property to lyse the bacterial cells and release the phage progeny, while lysogenic or temperate phages may go either through a lytic cycle or the phage genome may be integrated in the bacterial genome, constituting a prophage. Temperate phages contribute to the evolution of most bacteria, by promoting the transduction of various genes involved in virulence, fitness, and antibiotic resistance^7^. Even if in the human gut there are about 10^9^ virus-like particles per gram of faeces^8^, reports on *H. pylori* phages are still sparse. Moreover, the first *H. pylori* prophage was described almost 30 years after the discovery of *H. pylori*. During that long period, *H. pylori* was considered as a bacterium without prophages. Indeed the first *H. pylori* genomes sequenced did not reveal the presence of prophages, until the identification of a remnant prophage integrated into the genome of *H. pylori* strain B38^9^, confirmed later by the discovery of a larger prophage in strain B45^10^ and followed by other publications^11^,^12^. The prevalence of *H. pylori* prophages, inferred by the presence of the phage integrase gene, is estimated to be approximately 20%^10^ in *H. pylori* strains.

Prophages and bacteria are linked by a long history of co-evolution, but the genetic dimension of this co-evolution cannot be defined at present^7^. Indeed, a phylogenetic analysis of the integrase gene sequences present in *H. pylori* prophages revealed a strong phylogeographic signal within the phage integrase gene, which was in agreement with a model of co-evolution between the virus and its bacterial host. The presence of prophages in other non-*pylori Helicobacter* species, such as *Helicobacter acinonychis*^13^, *Helicobacter felis*^14^, or *Helicobacter bizzozeronii*^15^ which share homology with *H. pylori* prophages points to a prophage acquisition before speciation. The presence of remnant prophages (prophage fragments) in *H. pylori* strains^9^ and in non-*pylori* Helicobacters^16^, indicates a prophage decay during the complex interaction between *H. pylori* and the prophage. However, a model in which *H. pylori* strains from different geographical regions may have been infected by distinct phage lineages after the geographic separation of the bacterial host is also feasible^10^.

In order to understand if the prophage population structure coincides with its host structure, a group of 870 *H. pylori* strains were screened for the presence of prophages. Among them, 41 strains were positive for two prophage genes.

The *H. pylori* genomes and whole-genome shotgun (WGS) contigs databases were also Blast for the presence of the integrase and holin genes, allowing the identification of 22 *H. pylori* genomes which were also included in the study. These strains were selected and typed using the MLST method and a newly implemented method here designated as prophage sequence typing (PST), which targets the two prophage genes (integrase and holin) of *H. pylori*. A Bayesian clustering analysis was used for the identification of distinct genetic populations complemented by phylogenetic analysis, in order to determine the population structure of the host strains and their prophages. These approaches highlighted the diversity in the population structure of the *H. pylori* prophages. The present study demonstrates that the population structure analysis of *H. pylori* prophages discriminates between two different European populations while the traditional MLST-based method only distinguishes one.

## Results and Discussion

### Identification of *H. pylori* strains carrying prophages

The presence of prophages in *H. pylori* strains was confirmed by screening for 1) the integrase gene responsible for the integration of the phage genome into the bacterial chromosome, and 2) the holin gene involved in cell lysis when a lytic cycle occurs^7^. The integrase gene was previously shown to be a good marker for the presence of prophages^10^ and is usually placed at the left end, while the holin gene, is part of the lytic cassette and is usually placed on the right side. The presence of both genes may be indicative of intact prophages. Prophage sequences are highly heterogeneous, which can lead to false negative and positive PCR results. To overcome this problem, all PCR products were sequenced. The prophage sequences employed in the present study are available at GenBank (No: KM275873 to KM275935). The remaining integrase gene sequences were described previously^10^.

Due to their diversity it is possible that some prophages were not identified with these primer pairs. However, in light of the limited number of prophage sequences currently available, this approach was considered to be acceptable. Among the 870 *H. pylori* DNAs originally constituted, 161 (18.5%) were positive for the integrase gene and 41 (4.7%) for both the integrase and holin genes (Supplementary Table S2, strains 1 to 41).

The software for the identification of prophages^17^, applied to 53 *H. pylori* complete genomes listed at REBASE genomes^18^ in October 2014, revealed the presence of prophages in 18.9% (10/53) of the genomes. Only 5.6% (3/53) of these prophage positive genomes carried both the integrase and holin genes. These percentages are similar to those described in the present study. Moreover, a Blastn analysis^19^ of the integrase and holin genes using the nucleotide and the whole-genome shotgun contig databases allowed the identification of 22 genomes carrying these genes that were included in the analysis (Supplementary Table S1).

### *H. pylori*-prophage population structure

**Figure 1:**
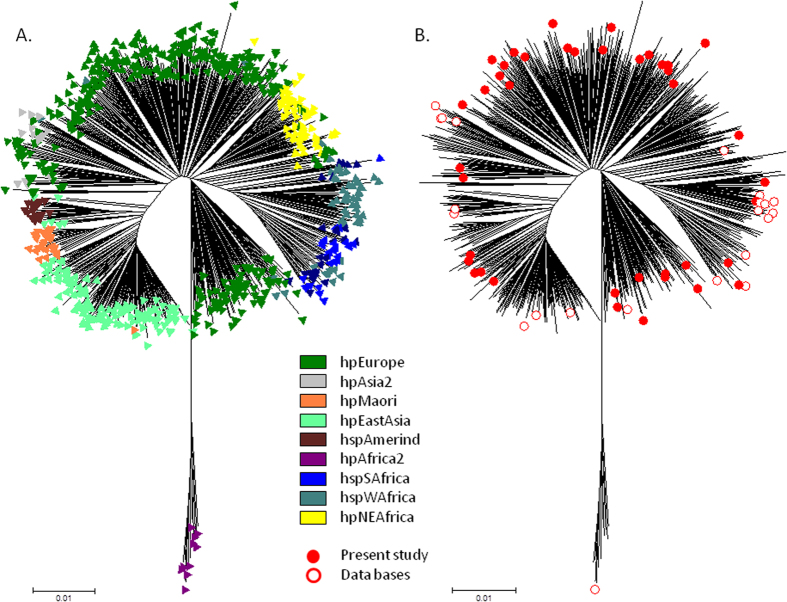
Neighbour-joining *H. pylori* MLST population tree of 804 strains. 741 strains from PubMLST (corresponding to initial studies by Falush *et al.*^1^ and Linz *et al.*^2^) and 63 strains from the present study. (**A**) The major *H. pylori* populations (six populations and three sub-populations) were identified according to the assigned population available at PubMLST. (**B**) Identification and position in the tree of the 41 strains and 22 genomes carrying the prophage integrase and holin genes.

After performing MLST using seven housekeeping genes, a phylogenetic tree was constructed including 741 concatenated sequences available at PubMLST for *H. pylori* (http://pubmlst.org/helicobacter/) and originally described by Falush *et al.*^1^ and Linz *et al.*^2^ plus 41 sequences from strains presenting the prophage genes and the 22 genomes previously selected (see above) (Fig. 1). The DNA sequences of the seven MLST genes are available at PubMLST (http://pubmlst.org/helicobacter/) with the profile numbers 2851 to 2886. The analysis shows that the strains included in the present study plus the genomes of *H. pylori* from public databases are dispersed throughout the major *H. pylori* populations.

For the Structure 2.3.4. analysis of the seven MLST genes of all 804 strains (741 from PubMLST, 41 presenting the prophage genes in the initial collection and 22 from public databases), the best posterior probabilities were achieved for K ≥ 6, but K > 6 produced inconsistent populations or hypothetical populations with no assigned individuals. Thus K = 6 was considered the best value. The major populations defined were consistent with prior assignments^2^, and were used to classify the 63 strains included in this study (Supplementary Table S2). Briefly, concerning the 41 selected strains, most were classified according to their country of origin (28 hpEurope strains, 9 hpAfrica1 strains and 4 hpEastAsia strains), with a few exceptions: one strain from France was classified as hpEastAsia and one strain from France and three strains from Portugal were classified as hpWAfrica or hpNEAfrica (Supplementary Table S2). Similar results were found for the 22 *H. pylori* genomes identified in public databases, i.e., when information was available, the population structure corresponded to the country of isolation, especially considering the diversity of isolates brought to the New World by non-Amerindian hosts^20^.

**Figure 2:**
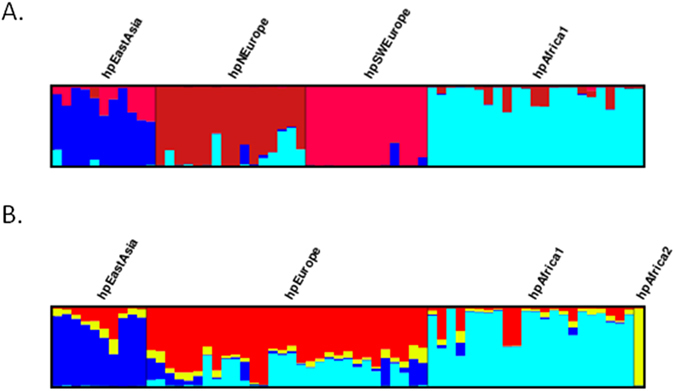
Distruct plot of Bayesian population assignments using STRUCTURE 2.3.4. and an admixture model (K = 4) for the 63 strains carrying prophages. (**A**) Distruct plot obtained with the prophage gene sequences (PST) and (**B**) with the sequences of seven housekeeping genes (MLST). Each bacterial isolate is depicted by a thin vertical line, which is divided into *K* coloured segments representing the membership coefficients in each cluster.

In the case of the Bayesian analyses of the seven MLST genes for the group of 63 strains carrying the prophage genes, the best posterior probability was achieved for K = 4, dividing the group into four populations: hpEurope (30 strains), hpEastAsia (10 strains), hpAfrica1 (22 strains) and hpAfrica2 (1 strain) (Fig. 2B). Using K = 5, a further hypothetical population with no assigned individuals was detected, and thus K = 4 fits these data the best. The same methodology was then applied to the prophage data. Surprisingly it produced four populations, since K = 4 presented the best posterior probability, showing two European populations. These four populations identified by PST (Fig. 2A) are hpEastAsia (11 strains), hpAfrica1 (23 strains) and two new European populations, hpSWEurope (13 strains) and hpNEurope (16 strains). The single strain comprising the hpAfrica2 population carries a prophage classified as hpNEurope. Briefly, the prophage sequences distinguished four populations with the parameters used, and the MLST sequences distinguished only three (not taking into account the hpAfrica2 strain, which appears to be a mosaic strain for the prophage sequence as explained below).

**Figure 3:**
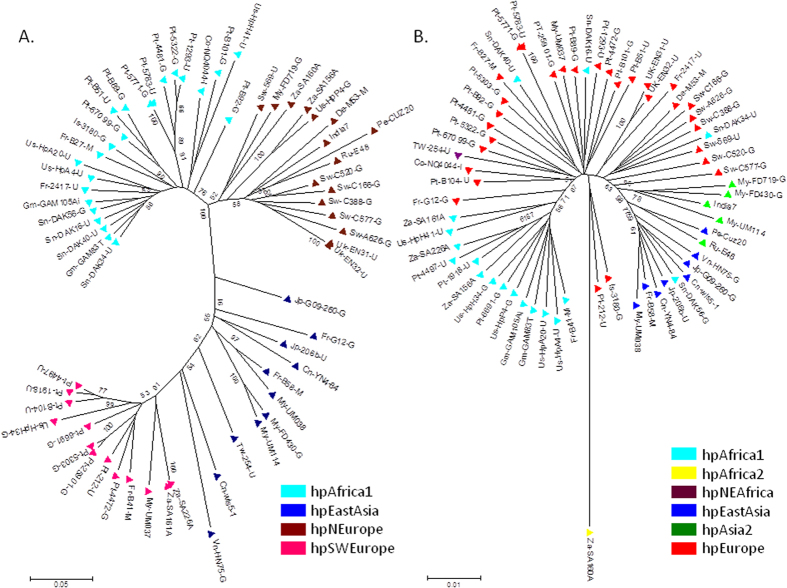
(**A**) Neighbour-joining tree (Kimura 2-parameter) of 63 concatenated prophage sequences from *H. pylori* strains carrying prophages colour-coded according to the population assigment by STRUCTURE using the prophage genes. (**B**) Neighbour-joining tree (Kimura 2-parameter) of 63 concatenated sequences obtained after MLST of *H. pylori* strains carrying prophages, and their attributed population structure after STRUCTURE analysis.

A tree for the 63 strains according to MLST is presented in Fig. 3B, including their population structure obtained after applying Structure 2.3.4.^21^,^22^,^23^ software. In both figures (Figs 1 and 3B.) there is a continuous distribution of the ancestry proportions, suggesting admixture due to recombination^2^.

A phylogenetic tree using the neighbour-joining method and the Kimura 2-parameter for the concatenated prophage genes showed that strains cluster according to their population assigned by Structure 2.3.4.^21^,^22^,^23^, in a continuous distribution (Fig. 3A). However, this distribution (Fig. 3A), is not as continuous as that observed in the *H. pylori* tree produced with the concatenated sequences of the seven housekeeping genes (Fig. 3B), revealing an increased diversity in the prophage gene sequences compared to the housekeeping genes. In fact, when only the sequences of the 63 strains were considered, among the 3,406 base pairs of the housekeeping genes, 824 positions were polymorphic, while among the 754 base pairs of the two prophage genes, 445 positions were polymorphic. The increased variability of *H. pylori* compared to its human host has been described and has allowed *H. pylori* to be used as a tool to trace human populations^1^. The prophage sequences could also be viewed as a tracer of human populations, allowing us to discriminate between two different European populations.

Falush *et al.* described the existence of two ancient European populations, AE1 and AE2, and considered that, according to MLST data, European isolates are recombinants of AE1 and AE2. AE1 is described as being higher in number in Northern Europe and Ladakh, while AE2 is higher in Southern Europe (Spain), Sudan and Israel^1^. AE1 is speculated to have arisen in Central Asia, while AE2 would had split from its sister lineage hpAfrica1, which then came in contact in Europe, forming the hybrid population hpEurope^24^. The European strains included in the present study confirm the mosaicism of small multiple chromosomal chunks observed after analysis of the seven housekeeping genes (Supplementary Fig. S1B). The genes included in the MLST for *H. pylori* are therefore not able to discriminate between current European populations, which have been attributed to multiple and complex migratory events that occurred over the European continent since its first colonization by modern men. However, considering the MLST analysis, a closer inspection of Fig. 3B shows that the hpNEurope strains from Fig. 3A cluster mostly in the right branch of the tree (the rare exceptions were hpAfrica2 strain Za-SA160A and two other mosaic strains Us-HpP4-G and Za-SA156A), whereas all hpSWEurope strains in Fig. 3A were found in the left branch of Fig. 3B. This was also evident in Fig. S1, where the hpSWEurope strains (in Table S2 with the numbers: 9, 21, 27, 29, 33, 35, 38, 39, 41, 44, 61, 62, 63) look quite different from the hpNEurope strains (in Table S2 with the numbers: 1, 2, 3, 4, 5, 6, 22, 23, 24, 42, 43, 45, 46, 47, 48, 49) according to MLST. Indeed, in Fig. S1 most of the strains classified as hpSWEurope by prophage typing are mosaics of hpEurope and hpAfrica1, while most of the strains considered hpNEurope according to prophage sequences, are in fact MLST mixtures of hpEurope and hpAsia2. Taken together these observations appear to indicate that the MLST sequences can weakly distinguish between *H. pylori* isolates from the north and southwest of Europe.

Surprisingly the analysis of the population structure according to the prophage genes of *H. pylori* discriminates between four populations, two of them from Europe showing little evidence of mosaicism between the Northern and Southwestern populations (Fig. 2). The primitive peoples that inhabited Europe, with their different migratory roots and languages (Germanic and Romance) could explain the existence of different European populations. The analysis of the prophage sequences can thus be a useful addition to MLST data to enable a discrimination between European populations.

Considering the mosaic structure of the European isolates according to MLST classification, an 84.1% concordance between the bacteria and the prophage genome population structure was observed (Supplementary Table S3). The discordant cases concern six strains (one French, three Portuguese, one South African and one North American), classified as hpAfrica1 according to bacterial genes and as hpSWEurope according to prophage genes; two strains (one French and one Portuguese) were both classified as hpEurope according to MLST and as hpAfrica according to PST; one Malaysian strain was classified as hpAsia2 by MLST and hpNEurope by PST (this particular strain appears to be a mosaic for MLST, i.e. a mixture between hpAsia2 and hpEastAsia, and a mosaic for PST, i.e. a mixture between hpNEurope and hpAfrica); and finally the single South African strain classified as hpAfrica2 by MLST and as hpNEurope by PST (this strain appears to be a mosaic for PST, a mixture of hpNEurope and hpAfrica). For the cases with no evidence of mosaicism, it is tempting to speculate that the prophage may have been recently acquired by horizontal gene transfer. Other isolates, classified differently according to one system or the other, were indeed mosaics. In fact, for these isolates the bacterial MLST or the PST showed evidence of a recombination with the population attributed by the other method, and these cases were thus considered as concordant classification. For instance, the strain Pt-5771-G showed a mosaicism between hpEurope and hpAfrica1 for the MLST genes and was classified as hpEurope by MLST and hpAfrica by PST (Supplementary Table S3). Taking into consideration the ancestral European populations, hpNEurope is most closely related to AE1 and hpSWEurope to AE2. Interestingly, most of the cases of discordant classification by MLST and PST concern exchanges between 1) MLST hpAfrica1 and PST hpSWEurope which appear to be closely related to AE2 which is a split from its sister lineage hpAfrica1 or 2) MLST hpAsia2 and PST hpNEurope, closely related to AE1, which have probably arisen from Central Asia. The segregation observed in European populations is in accordance with recent human genetic data which can place individuals in different European localizations using 40,000–130,000 single nucleotide polymorphisms (SNP)^25^,^26^. Moreover, the segregation of European populations observed in the prophage gene analysis appears to be in agreement with the most recent data concerning ancestral populations of Europe, influenced by the ancient people of Western Europe, Northern Eurasia and the Near East^27^.

In summary, our results show the existence of different European populations which could be traced using dormant phages of *H. pylori*. Thus PST is able to differentiate individual populations within the European population, which is in agreement with recent human genome-wide analysis favouring the hypothesis that present-day European genetic diversity is strongly correlated with geography^27^,^28^.

## Methods

### *H. pylori* strains

Genomic DNA samples from 870 *H. pylori* strains, isolated from patients living in different continents (692 were from Europe, 117 from Asia, 1 from the USA and 60 from Africa) and suffering from different diseases (249 peptic ulcer, 450 gastritis, 77 adenocarcinoma, and 65 MALT lymphoma; information concerning the associated disease was lacking for 29 strains) were collected. These DNAs were extracted using standard protocols after culture of *H. pylori* strains belonging to the collection of the French National Reference Centre for Campylobacters and Helicobacters (F. Mégraud and P. Lehours, Bordeaux, France); the Department of Microbiology, Tumor and Cell Biology, Karolinska Institute (Lars Engstrand); the Klinikum Rechts Der Isar II, Medical Department, Technische Universität, Munich, Germany (M. Gerhard); the Department of Medicine-Gastroenterology, Michael E. DeBakey Veterans Affairs Medical Center and Baylor College of Medicine, Houston, TX, USA (Y. Yamaoka); the Department of Infectious Diseases, National Institute of Health, Lisbon, Portugal (M. Oleastro); the Rabin Medical Center – Beilinson Hospital, Petah Tikva, Israel (T.T. Perets and Y. Niv); and the Pasteur Institute of Dakar, Senegal (S. Breurec).

From this initial group of *H. pylori* genomic DNA samples, 41 strains (Supplementary Table S2, strains 1 to 41) were selected for the presence of the target prophage genes, integrase and holin. These strains were then typed by MLST; for this purpose, seven housekeeping genes were amplified and sequenced, as previously described^1^.

The *H. pylori* genomes and WGS available at public databases were subjected to Blastn analysis^19^ for the presence of the integrase and holin prophage genes, with a threshold limit of <1e^−6^. The genomes carrying these genes were selected if isolated from human hosts. In the cases of multiple isolates from the same patient, only one genome was considered. In the selected genomes the two prophage genes and the seven MLST gene sequences were collected for further analysis along with the gene sequences of the 41 strains.

### Implementation of a prophage sequence typing (PST) scheme for the prophages of *H. pylori*

Two prophage genes were selected to be used for the PST; integrase and holin. All strains were tested for the presence of integrase and positive strains were also tested for the presence of holin.

The collection of genomic DNA from *H. pylori* was first challenged by PCR for the presence of the integrase gene of the prophage, using the degenerated primers F1, AAGYTTTTTAGMGTTTTGYG, and R1, CGCCCTGGCTTAGCATC (Eurofins Genomics, Ebersberg, Germany) which produce a 529-bp PCR product^10^. The strains carrying the integrase gene were then tested for the presence of holin. Degenerated primers based on the holin gene sequences of phages 1961P, KHP30, KHP40 and *H. pylori* strains Cuz20, India7 and *H. acynonychis* were used. The primers were hol-F CCATCCCGTATTTGTTGGTG and hol-R ACCCAATGCCTCCACTAATC (Eurofins Genomics) producing a 225-bp PCR product. In both cases, the PCR mix included Promega (Madison, Wisconsin, USA) buffer (1X), dNTPs (0.2 μM), primers (0.5 μM each), GoTaq polymerase (1.5 U), water to complete 25 μl and DNA sample (25 to 50 ng). The PCR cycle was composed of a first cycle at 95 °C for 4 minutes, 35 cycles at 95 °C for 30 seconds, 58 °C for 30 seconds and 72 °C for 1 minute. A last cycle at 72 °C for 7 minutes was applied.

The PCR products of each positive strain were purified using MicroSpin S-400 or S-300HR columns (GE Healthcare, Saclay, France) and directly sequenced on both strands using The BigDye® Terminator v3.1 Cycle Sequencing Kits (Applied Biosystems, Villebon-sur-Yvette, France), using an ABI 3700 analyzer DNA sequencer (PE Applied Biosystems).

To validate the reproducibility of this approach to identify prophages, a software for prophage detection^17^ in 53 complete *H. pylori* genomes (listed at REBASE genomes^18^ and retrieved from the NCBI in October 2014) was used.

### *H. pylori* MLST and PST data analysis

The Structure 2.3.4.^21^,^23^,^29^ program was used to study the number of populations K using the admixture model for the *H. pylori* MLST and for PST. All gene sequences were first aligned and the file was converted to the STRUCTURE input file using xmfa2structure by X. Didelot and D. Falush (http://www.xavierdidelot.xtreemhost.com/clonalframe.htm). All runs were performed in duplicate. In each run, a Markov Chain Monte Carlo (MCMC) of 10,000 iterations and a burn-in period of 10,000 iterations were chosen. The highest mean value of ln likelihood was compared for multiple runs of 2 ≤ K ≤ 9 to select the K value that fit the best. For the *H. pylori* MLST analysis we included 741 other MLST profiles available on the PubMLST website (http://pubmlst.org/helicobacter/) taking into consideration previously used profiles^1^,^2^. The Structure 2.3.4. was also used, under the same conditions, for the analysis of the 41 strains plus 22 genomes (identified in public databases) after *H. pylori* MLST alone or with PST.

The trimmed and concatenated sequences of the seven *H. pylori* MLST genes from 741 strains available at PubMLST plus 41 strains included in this study and 22 genomes from public databases were aligned using MAFFT version7^30^ and were used for the construction of a phylogenetic tree. A neighbour-joining phylogenetic tree topology of nucleotide alignments was constructed using the MEGA (Molecular Evolutionary Genetics Analysis) 6.0 software^31^, on the basis of distances estimated using the Kimura two-parameter model^32^. Branching significance was estimated using bootstrap confidence levels by randomly resampling the data 1,000 times with the referred evolutionary distance model. A phylogenetic tree was also constructed using only the sequences of the 41 strains included in the present work plus the sequences of the 22 genomes available in public databases. A similar approach was used for the construction of a phylogenetic tree of the trimmed and concatenated prophage genes, after alignment and elimination of poorly aligned positions and divergent regions of the alignment of DNA sequences using Gblocks^33^. These positions may not be homologous or may have been saturated by multiple substitutions and it is convenient to eliminate them prior to phylogenetic analysis.

## Additional Information

**Accession codes**: The DNA sequences of the seven MLST genes are available at the PubMLST website (http://pubmlst.org/helicobacter/) with the profile numbers 2851 to 2886. The prophage sequences employed in the present study are available at GenBank (No: KM275873 to KM275935).

**How to cite this article**: Vale, F. F. *et al.* Dormant phages of *Helicobacter pylori* reveal distinct populations in Europe. *Sci. Rep.* **5**, 14333; doi: 10.1038/srep14333 (2015).

## Supplementary Material

Supplementary Information
