# Supplementary material for: Dormant phages of Helicobacter pylori reveal distinct populations in Europe
Source: Sci Rep. 2015 Sep 21;5:14333. doi: 10.1038/srep14333 (PMC4585682; doi:10.1038/srep14333)
Supplement: Supplementary Information [file srep14333-s1.pdf]

Dormant phages of *Helicobacter pylori* reveal distinct populations in Europe

F. F. Vale, J. Vadivelu, M. Oleastro, S. Breurec, L. Engstrand, T. T. Perets, F. Mégraud, P. Lehours

Supplementary Information

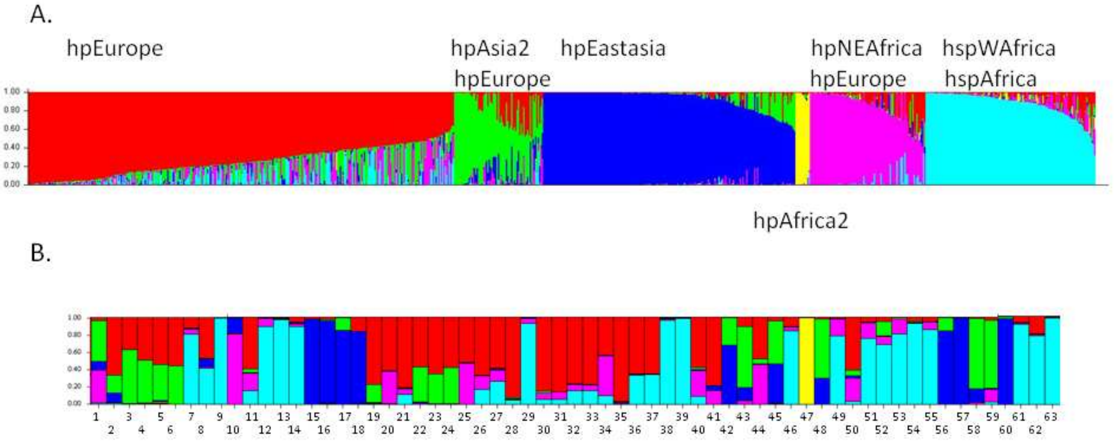

Fig S1. Bayesian population assignments using STRUCTURE 2.3.4. A. Plot of the population assignment of 741 sequences available at PubMLST plus an additional 63 sequences of strains carrying prophages using an admixture model (*K*=6). Each bacterial isolate is depicted by a thin vertical line, which is divided into *K* coloured segments representing the membership coefficients in each cluster. The identification of the population attributed to the strains according to information available at PubMLST is found above and below the figure. B. Detailed plot of the strains carrying prophages (strain numbers above are depicted in Supplementary Table S2).

Table S1. *Helicobacter pylori* genomes carrying prophage integrase and holin genes retrieved from public databases.

Genome	BLAST Database	BLAST results				Observation
		Integrase		Holin		
		Query cov.	E-value	Query cov.	E-value	
Cuz20	nucleotide collection	95%	$2e^{-143}$	90%	$2e^{-40}$	
E48	WGS	95%	$2e^{-163}$	100%	$1e^{-61}$	
FD430	WGS	80%	$9e^{-86}$	91%	$4e^{-54}$	
FD719	WGS	97%	$5e^{-139}$	99%	$1e^{-55}$	
GAM105Ai	WGS	97%	$5e^{-120}$	96%	$1e^{-42}$	
GAM83T	WGS	97%	$3e^{-123}$	94%	$5e^{-40}$	Minor differences in relation to genome GAM83Bi, which was not included. No information available but probably isolates from the same individual
H-34H	WGS	100%	$5e^{-51}$	100%	$2e^{-57}$	
Hp A-20	WGS	97%	$4e^{-115}$	96%	$2e^{-44}$	
Hp A-4	WGS	97%	$2e^{-119}$	93%	$4e^{-35}$	
Hp H-41	WGS	98%	$1e^{-122}$	91%	$2e^{-38}$	
Hp P-4	WGS	97%	$1e^{-135}$	100%	$6e^{-71}$	Minor differences in relation to genomes Hp P-4c and Hp P-4d, which were not included. No information available but probably isolates from the same individual
India7	nucleotide collection	98%	$1e^{-165}$	100%	$1e^{-60}$	
NQ4044NQ	WGS	98%	$9e^{-124}$	96%	$5e^{-59}$	
NY4-84	WGS	98%	$4e^{-96}$	96%	$1e^{-61}$	
PZ5080	WGS	100%	$2e^{-49}$	96%	$5e^{-40}$	The complete *atpA* gene sequence was not found. Not included.
SA156A	WGS	97%	$9e^{-143}$	100%	$5e^{-59}$	Identical to the genome SA156C that was isolated from the same individual. Not included.
SA160A	WGS	97%	$4e^{-141}$	100%	$6e^{-52}$	Identical to the genome SA60C that was isolated from the same individual. Not included.
SA161A	WGS	83%	$3e^{-54}$	100%	$2e^{-57}$	Identical to the genome SA161C that was isolated from the same individual. Not included.
SA226A	WGS	100%	$2e^{-55}$	100%	$3e^{-56}$	
UM037	nucleotide collection	100%	$3e^{-53}$	92%	$2e^{-59}$	
UM038	WGS	98%	$6e^{-94}$	91%	$2e^{-46}$	
UM114	WGS	95%	$3e^{-85}$	100%	$6e^{-71}$	
wls-5-1	WGS	100%	$3e^{-66}$	100%	$3e^{-56}$	The genomes wls-5-1 to wls-5-18 also carry the genes, but correspond to isolates from the same individual. Only wls-5-1 was included.

WGS - whole-genome shutgun contigs; Query cov. - query coverage.

Table S2. *Helicobacter pylori* strains carrying integrase and holin genes.

#	Strain	Country of isolation	Associated disease	Population structure (most probable population)	
				H. pylori MLST	PST
1	Sw-C577-G	Sweden	Gastritis	hpEurope	hpNEurope
2	Sw-C520-G	Sweden	Gastritis	hpEurope	hpNEurope
3	Sw-C388-G	Sweden	Gastritis	hpEurope	hpNEurope
4	De-M53-M	Germany	MALT lymphoma	hpEurope	hpNEurope
5	Sw-C166-G	Sweden	Gastritis	hpEurope	hpNEurope
6	Sw-A626-G	Sweden	Gastritis	hpEurope	hpNEurope
7	Sn-DAK16-U	Senegal	Peptic ulcer	hspWAfrica	hpAfrica1
8	Fr-G12-G	France	Gastritis	hpEurope	hpEastAsia
9	Fr-B41-M	France	MALT lymphoma	hspWAfrica	hpSWEurope
10	Tw-254-U	Taiwan	Peptic ulcer	hpNEAfrica	hpEastAsia
11	Fr-B27-M	France	MALT lymphoma	hpEurope	hpAfrica1
12	Sn-DAK40-U	Senegal	Peptic ulcer	hspWAfrica	hpAfrica1
13	Sn-DAK34-U	Senegal	Peptic ulcer	hspWAfrica	hpAfrica1
14	Sn-DAK56-G	Senegal	Gastritis	hspWAfrica	hpAfrica1
15	Jp-206b-U	Japan	Peptic ulcer	hpEastAsia	hpEastAsia
16	Fr-B58-M	France	MALT lymphoma	hpEastAsia	hpEastAsia
17	Jp-G09-260-G	Japan	Gastritis	hpEastAsia	hpEastAsia
18	Vn-HN75-G	Vietnam	Gastritis	hpEastAsia	hpEastAsia
19	Fr-2417-U	France	Peptic ulcer	hpEurope	hpAfrica1
20	Pt-B101-G	Portugal	Gastritis	hpEurope	hpAfrica1
21	Pt-B104-U	Portugal	Peptic ulcer	hpEurope	hpSWEurope
22	Sw-569-U	Sweden	Peptic ulcer	hpEurope	hpNEurope
23	Uk-EN31-U	United Kingdom	Peptic ulcer	hpEurope	hpNEurope
24	Uk-EN32-U	United Kingdom	Peptic ulcer	hpEurope	hpNEurope
25	Pt-5322-G	Portugal	Gastritis	hpEurope	hpAfrica1
26	Pt-4481-G	Portugal	Gastritis	hpEurope	hpAfrica1
27	Pt-212-U	Portugal	Peptic ulcer	hpEurope	hpSWEurope
28	Pt-1293-U	Portugal	Peptic ulcer	hpEurope	hpAfrica1
29	Pt-1918-U	Portugal	Peptic ulcer	hspWAfrica	hpSWEurope
30	Pt-B51-U	Portugal	Peptic ulcer	hpEurope	hpAfrica1
31	Pt-B89-G	Portugal	Gastritis	hpEurope	hpAfrica1
32	Pt-B92-G	Portugal	Gastritis	hpEurope	hpAfrica1
33	Pt-259_01-G	Portugal	Gastritis	hpEurope	hpSWEurope
34	Pt-670_99-G	Portugal	Gastritis	hpEurope	hpAfrica1
35	Pt-5303-G	Portugal	Gastritis	hpEurope	hpSWEurope
36	Pt-5771-G	Portugal	Gastritis	hpEurope	hpAfrica1
37	Pt-5783-U	Portugal	Peptic ulcer	hpEurope	hpAfrica1
38	Pt-6691-G	Portugal	Gastritis	hspWAfrica	hpSWEurope
39	Pt-4497-U	Portugal	Peptic ulcer	hspWAfrica	hpSWEurope
40	Il-3180-G	Israel	Gastritis	hpEurope	hpAfrica1
41	Pt-4472-G	Portugal	Gastritis	hpEurope	hpSWEurope

42	Pe-Cuz20	Peru	na	hpEastAsia	hpNEurope
43	India7	na	na	hpAsia2	hpNEurope
44	My-UM037	Malaysia	na	hpEurope	hpSWEurope
45	Ru-E48	Russia	na	hpAsia2	hpNEurope
46	Za-SA156A	South Africa	na	hspSAfrica	hpNEurope
47	Za-SA160A	South Africa	na	hpAfrica2	hpNEurope
48	My-FD719-G	Malaysia	Gastritis	hpAsia2	hpNEurope
49	Us-HpP4-G	USA	Gastritis	hpAfrica1	hpNEurope
50	Co-NQ4044-I	Colombia	Intestinal metaplasia	hpEurope	hpAfrica1
51	Gm-GAM83T	The Gambia	na	hspWAfrica	hpAfrica1
52	Us-HpH41-U	USA	Peptic ulcer	hpAfrica1	hpAfrica1
53	Gm-GAM105Ai	The Gambia	na	hspWAfrica	hpAfrica1
54	Us-HpA4-U	USA	Peptic ulcer	hpAfrica1	hpAfrica1
55	Us-HpA20-U	USA	Peptic ulcer	hpAfrica1	hpAfrica1
56	Cn-YN4-84	China	na	hpEastAsia	hpEastAsia
57	My-UM038	Malaysia	na	hpEastAsia	hpEastAsia
58	My-FD430-G	Malaysia	Gastritis	hpAsia2	hpEastAsia
59	My-UM114	Malaysia	na	hpAsia2	hpEastAsia
60	Cn-wls5-1	China	na	hpEastAsia	hpEastAsia
61	Za-SA226A	South Africa	na	hspWAfrica	hpSWEurope
62	Za-SA161A	South Africa	na	hspWAfrica	hpSWEurope
63	Us-HpH-34-G	USA	Gastritis	hpAfrica1	hpSWEurope

na - information not available, MLST – multilocus sequence typing, PST - prophage sequence typing, MALT - mucosa associated lymphoid tissue.

Table S3. Inferred ancestry of individuals according to the Bayesian analysis of *Helicobacter pylori* strains using Structure 2.3.4. The most probable populations are highlighted in grey.

MLST						Strains	PST			
hpEurope	hpAsia2 hpEurope	hspMaori hspEAsia hspAmerind	hpAfrica2	hpNEAfrica hpEurope	hspWAfrica1 hspSAfrica1		hpAfrica	hpEAsia	hpNEurope	hpSWEurope
0.031	**0.475**	0.095	0.003	0.381	0.015	Sw-C577-G	0.005	0.001	**0.993**	0.001
**0.664**	0.205	0.109	0.001	0.003	0.019	Sw-C520-G	0.197	0.001	**0.801**	0.001
0.369	**0.622**	0.002	0.001	0.002	0.003	Sw-C388-G	0.007	0.003	**0.989**	0.001
0.491	**0.493**	0.002	0.006	0.007	0.001	De-M53-M	0.018	0.003	**0.978**	0.002
**0.536**	0.421	0.014	0.009	0.007	0.013	Sw-C166-G	0.002	0.001	**0.996**	0.001
**0.554**	0.440	0.001	0.001	0.002	0.002	Sw-A626-G	0.006	0.002	**0.991**	0.001
0.121	0.005	0.002	0.001	0.062	**0.810**	Sn-DAK16-U	**0.996**	0.001	0.002	0.001
**0.465**	0.011	0.093	0.001	0.010	0.420	Fr-G12-G#	0.203	**0.710**	0.001	0.086
0.001	0.001	0.001	0.000	0.002	**0.995**	Fr-B41-M*	0.001	0.003	0.003	**0.993**
0.003	0.002	0.179	0.003	**0.809**	0.003	Tw-254-U#	0.001	**0.762**	0.001	0.235
**0.590**	0.043	0.001	0.002	0.205	0.159	Fr-B27-M#	**0.901**	0.015	0.081	0.003
0.006	0.004	0.003	0.001	0.092	**0.894**	Sn-DAK40-U	**0.991**	0.001	0.007	0.001
0.007	0.001	0.004	0.006	0.004	**0.978**	Sn-DAK34-U	**0.996**	0.001	0.001	0.002
0.052	0.001	0.016	0.003	0.033	**0.895**	Sn-DAK56-G	**0.996**	0.001	0.001	0.001
0.018	0.003	**0.974**	0.000	0.002	0.002	Jp-206b-U	0.002	**0.989**	0.004	0.005
0.026	0.015	**0.944**	0.002	0.008	0.004	Fr-B58-M	0.001	**0.970**	0.001	0.028
0.003	0.140	**0.855**	0.000	0.001	0.001	Jp-G09-260-G	0.075	**0.784**	0.135	0.005
0.158	0.003	**0.830**	0.001	0.004	0.004	Vn-HN75-G	0.004	**0.634**	0.007	0.355
**0.769**	0.208	0.004	0.001	0.009	0.009	Fr-2417-U*	**0.967**	0.002	0.031	0.001
**0.611**	0.003	0.002	0.001	0.373	0.010	Pt-B101-G#	**0.773**	0.005	0.220	0.002
**0.806**	0.003	0.006	0.000	0.066	0.119	Pt-B104-U	0.001	0.001	0.001	**0.997**
**0.569**	0.397	0.015	0.002	0.010	0.006	Sw-569-U	0.403	0.005	**0.588**	0.003
**0.650**	0.341	0.004	0.001	0.003	0.001	Uk-EN31-U	0.001	0.001	**0.998**	0.001
**0.576**	0.415	0.002	0.000	0.005	0.001	Uk-EN32-U	0.001	0.001	**0.998**	0.001
**0.519**	0.001	0.002	0.001	0.471	0.006	Pt-5322-G#	**0.995**	0.001	0.003	0.001
**0.662**	0.001	0.003	0.002	0.154	0.178	Pt-4481-G#	**0.677**	0.003	0.319	0.001
**0.580**	0.007	0.015	0.005	0.127	0.265	Pt-212-U	0.001	0.002	0.001	**0.996**
**0.918**	0.016	0.007	0.001	0.004	0.053	Pt-1293-U*	**0.997**	0.001	0.001	0.001
0.007	0.001	0.002	0.000	0.061	**0.928**	Pt-1918-U*	0.001	0.001	0.000	**0.998**
**0.848**	0.020	0.002	0.001	0.070	0.059	Pt-B51-U#	**0.982**	0.002	0.015	0.001
**0.851**	0.001	0.001	0.001	0.083	0.064	Pt-B89-G#	**0.753**	0.001	0.245	0.001
**0.760**	0.006	0.003	0.001	0.070	0.161	Pt-B92-G#	**0.751**	0.001	0.246	0.001
**0.766**	0.002	0.003	0.001	0.066	0.163	Pt-259_01-G	0.000	0.001	0.001	**0.998**
0.431	0.002	0.003	0.001	**0.459**	0.104	Pt-670_99-G#	**0.994**	0.002	0.003	0.001
**0.962**	0.007	0.006	0.001	0.015	0.011	Pt-5303-G	0.000	0.001	0.001	**0.998**
**0.646**	0.004	0.003	0.001	0.006	0.341	Pt-5771-G#	**0.997**	0.001	0.001	0.001
**0.646**	0.004	0.002	0.001	0.002	0.345	Pt-5783-U#	**0.997**	0.001	0.001	0.001
0.006	0.001	0.001	0.010	0.013	**0.969**	Pt-6691-G*	0.001	0.001	0.001	**0.997**
0.004	0.001	0.001	0.001	0.004	**0.989**	Pt-4497-U*	0.001	0.001	0.000	**0.998**
**0.575**	0.024	0.009	0.001	0.295	0.096	Il-3180-G#	**0.896**	0.020	0.083	0.001
**0.781**	0.003	0.056	0.001	0.155	0.006	Pt-4472-G	0.001	0.005	0.002	**0.992**

0.004	0.315	**0.678**	0.000	0.001	0.001	Pe-Cuz20#	0.019	0.250	**0.730**	0.001
0.102	**0.703**	0.146	0.001	0.044	0.003	India7#	0.004	0.011	**0.979**	0.006
**0.473**	0.062	0.003	0.001	0.454	0.006	My-UM037	0.001	0.290	0.010	**0.699**
0.040	**0.495**	0.449	0.004	0.006	0.006	Ru-E48#	0.095	0.043	**0.861**	0.001
0.101	0.005	0.003	0.001	0.042	**0.848**	Za-SA156A#	0.167	0.003	**0.829**	0.001
0.001	0.000	0.000	**0.997**	0.001	0.001	Za-SA160A*/#	0.435	0.027	**0.525**	0.012
0.019	**0.679**	0.295	0.001	0.003	0.003	My-FD719-G*	0.481	0.014	**0.503**	0.001
0.012	0.003	0.001	0.001	0.192	**0.790**	Us-HpP4-G#	0.206	0.001	**0.792**	0.001
**0.607**	0.059	0.031	0.001	0.262	0.040	Co-NQ4044-I#	**0.870**	0.003	0.082	0.045
0.054	0.002	0.002	0.001	0.182	**0.760**	Gm-GAM83T	**0.995**	0.002	0.002	0.001
0.048	0.161	0.003	0.004	0.095	**0.689**	Us-HpH41-U	**0.712**	0.007	0.280	0.001
0.004	0.001	0.001	0.010	0.174	**0.810**	Gm-GAM105Ai	**0.995**	0.001	0.003	0.001
0.040	0.013	0.001	0.006	0.008	**0.932**	Us-HpA4-U	**0.979**	0.003	0.017	0.001
0.049	0.002	0.002	0.001	0.082	**0.865**	Us-HpA20-U	**0.982**	0.001	0.016	0.001
0.002	0.155	**0.840**	0.000	0.001	0.001	Cn-YN4-84	0.003	**0.837**	0.006	0.153
0.001	0.001	**0.996**	0.001	0.001	0.000	My-UM038	0.002	**0.987**	0.002	0.009
0.010	**0.811**	0.156	0.001	0.021	0.002	My-FD430-G#	0.001	**0.677**	0.003	0.319
0.029	**0.779**	0.018	0.001	0.143	0.030	My-UM114#	0.001	**0.579**	0.002	0.418
0.004	0.019	**0.975**	0.001	0.001	0.001	Cn-wls5-1	0.011	**0.552**	0.002	0.435
0.070	0.003	0.004	0.001	0.014	**0.908**	Za-SA226A*	0.001	0.002	0.001	**0.997**
0.196	0.002	0.003	0.001	0.016	**0.783**	Za-SA161A#	0.001	0.002	0.001	**0.997**
0.007	0.002	0.002	0.001	0.006	**0.982**	Us-HpH-34-G*	0.001	0.106	0.001	**0.893**

* Strains with probably more recently acquired prophages (no coincidence in the population structure). # Mosaic structure of MLST classification in agreement with PST classification or vice-versa.
MLST – multilocus sequence typing, PST - prophage sequence typing
